# Matched-pair analysis of motor outcomes in adults with spinal muscular atrophy on nusinersen vs. risdiplam

**DOI:** 10.1007/s00415-025-13589-w

**Published:** 2026-01-03

**Authors:** Svenja Neuhoff, Benjamin Stolte, Jaqueline Lipka, Melina Schlag, Refik Pul, Linda-Isabell Schmitt, Markus Leo, Jelena Skuljec, Cornelius Deuschl, Michael Forsting, Christoph Kleinschnitz, Tim Hagenacker

**Affiliations:** 1 Department of Neurology and Center for Translational Neuro- and Behavioral Sciences (C-TNBS), University Hospital Essen, Hufelandstr. 55, 45147 Essen, Germany; 2 Institute for Diagnostic and Interventional Radiology and Neuroradiology, University Medicine Essen, University of Duisburg-Essen, Essen, Germany

**Keywords:** Spinal muscular atrophy, Nusinersen, Risdiplam, Matched-pair analysis, Comparison, Motor outcome

## Abstract

**Background:**

Nusinersen and risdiplam are approved disease-modifying therapies for adults with 5q-associated spinal muscular atrophy (SMA). To date, no direct comparison of the two treatments in adults has been conducted. Real-world cohorts of nusinersen and risdiplam differ in key baseline characteristics, such as motor function and disease severity, making direct comparison challenging. Nevertheless, such analyses are important for treatment decisions.

**Methods:**

We conducted a single-center, prospective, matched-pair analysis of adult persons with SMA (pwSMA) treated with nusinersen or risdiplam between 2017 and 2025. Patients were matched 1:1 based on baseline motor scores (Hammersmith Functional Motor Scale-Expanded [HFMSE], Revised Upper Limb Module [RULM]) and adjusted for age and disease duration at treatment initiation. Motor function was assessed at baseline, 4–8, 10–14, 22–26, and 32–40 months after treatment initiation. Pairwise difference scores (Δ-values) were analyzed using non-parametric tests.

**Results:**

From a cohort of 101 pwSMA (65 nusinersen, 36 risdiplam), 24 matched pairs (*n* = 48) were identified. Baseline demographic and clinical characteristics did not differ between groups. Over a maximum follow-up of nearly three years, no differences were observed in the trajectories of HFMSE or RULM scores between the nusinersen and risdiplam group. Within each group, motor function remained stable without significant decline.

**Conclusions:**

In this first matched-pair comparison of nusinersen and risdiplam in adults with SMA, both treatments achieved similar stabilization of motor function over almost three years. Larger, multicenter studies are warranted to confirm these results and explore potential subgroup-specific treatment effects.

**Supplementary Information:**

The online version contains supplementary material available at 10.1007/s00415-025-13589-w.

## Background

5q-associated spinal muscular atrophy (SMA) is a genetic disorder that is primarily characterized by dysfunction and degeneration of lower motor neurons, leading to progressive muscle weakness and atrophy in the limbs, bulbar region, and respiratory muscles [1–3]. The underlying cause is a homozygous deletion or a combination of deletion and point mutation in the *SMN1* (survival of motor neuron 1) gene, resulting in an SMN protein deficiency [1]. The *SMN2* (survival of motor neuron 2) gene, which is present in variable copy number, produces only a small amount of functional SMN protein due to alternative splicing and therefore offers only limited compensation [4]. The phenotype of SMA is largely determined by the number of *SMN2* copies [5]. Individuals with a low number of *SMN2* copies, as seen in SMA types 1 and 2, typically develop severe symptoms in early childhood. In contrast, individuals with higher copy numbers, as in SMA types 3 and 4, often show a later onset of symptoms in adolescence or adulthood and tend to have a milder disease course [5]. Today, individuals with SMA are additionally classified functionally according to their current best motor function in non-sitters, sitters, and walkers [6].

Over the past decade, gene therapeutic options have transformed the treatment landscape of SMA. Nusinersen, an intrathecally administered antisense oligonucleotide, and risdiplam, an orally administered small molecule, are both approved for the treatment of adults with SMA [7]. Both therapies modify the splicing of *SMN2* pre-mRNA, increasing the production of functional SMN protein [8, 9]. Both have demonstrated stabilization or improvement in motor function even in adults with an advanced disease stage in real-world studies [10–14]. In clinical practice, the choice between the two treatments is guided by practical aspects like patient preference, the technical feasibility of intrathecal administration, or national reimbursement situations. To date, no direct comparison of the effectiveness of nusinersen and risdiplam in adults has been conducted. One major obstacle is the difficulty of comparing treatment groups with differing baseline motor function—an important factor, since individuals with better baseline motor status often show greater improvement under therapy [10, 11]. The difference in baseline motor function between the two treatment groups is primarily due to the fact that patients with severe neuromuscular scoliosis are preferably treated with risdiplam [15]. Consequently, adults receiving risdiplam are on average more severely affected and predominantly classified as SMA type 2, whereas those treated with nusinersen tend to be less impaired and are mostly classified as SMA type 3. This difference has been consistently observed in real-world studies [10–14].

While comparative data on the effectiveness of nusinersen and risdiplam from real-world studies are now available for children with SMA—showing no superiority of either treatment in SMA types 2 and 3, but indicating an advantage of risdiplam in type 1—no such data are available for adults with SMA [16–18]. Nonetheless, a direct comparison of both treatments in adults is of considerable scientific and clinical relevance. It may yield insights into the differential effects of administration routes and drug distribution, thereby contributing to the optimization and further development of SMA therapies. Moreover, a comparison can support evidence-based decision-making by both clinicians and patients. In our single-center study, we aimed to perform a direct and transparent comparison of the effects of nusinersen and risdiplam on motor function in adults with SMA by applying a matched-pair analysis with statistical adjustments to account for baseline differences.

## Methods

### Study design and participants

This study was conducted in the Department of Neurology, University Hospital, Essen, Germany. Participants provided written informed consent prior to their inclusion in the study. The study was approved by the local ethics committee of the University of Duisburg-Essen, Germany (approval number 18–8071-BO). We included adult individuals with molecularly confirmed 5q-associated spinal muscular atrophy, referred to as persons with SMA (pwSMA) defined by a homozygous deletion of the *SMN1* gene or compound heterozygosity for a deletion and a point mutation in *SMN1*. All pwSMA were treated with either nusinersen or risdiplam in accordance with the approved label for a minimum of 4 months and were prospectively evaluated every 4 months. In patients with spondylodesis, nusinersen was administered via CT-guided lumbar puncture. At each visit, the Hammersmith Functional Motor Scale-Expanded (HFMSE) and Revised Upper Limb Module (RULM) were evaluated. Both are established and validated outcome measures for assessing motor function in adults with SMA [19, 20]. The HFMSE consists of 33 items scored 0–2, with a maximum score of 66, and the RULM comprises 19 items with a maximum score of 37. Higher scores indicate better motor function. Additionally, the following clinical and demographic parameters were recorded: sex, age, disease duration until treatment initiation, SMA type, *SMN2* copy number, clinical phenotype, presence of a percutaneous endoscopic gastrostomy (PEG), ventilatory support, and spondylodesis. Data collection took place between 2017 and 2025. Five assessment time points were defined: baseline (T0), 4–8 months (T1), 10–14 months (T2), 22–26 months (T3), and 32–40 months (T4) after treatment initiation. PwSMA who had previously received another SMN-targeting therapy and those without baseline HFMSE and RULM scores were excluded from analysis. Using rule-based matching and a distance score, pairs of patients treated with nusinersen or risdiplam were formed based on similarity in motor baseline function as well as relevant clinical and demographic characteristics. The course of motor function was then compared within these matched pairs.

### Matching and statistical analysis

A rule-based 1:1 matching with a distance score was performed in R (R Core Team version 4.5.0). From the total cohort (*n* = 101), all potential nusinersen candidates were first identified for each risdiplam-treated patient, based on a maximum difference of one point in both baseline HFMSE and baseline RULM scores. For each eligible candidate pair, a matching score was calculated. This score incorporated differences in motor function (baseline HFMSE and RULM) as well as age at treatment initiation and disease duration until treatment initiation. Functional differences (HFMSE and RULM) were fully weighted, while demographic variables were weighted by a factor of 0.2 to reduce their dominance but still include their influence. The matching score was therefore defined as:

Δ HFMSE + Δ RULM + 0.2 × (Δ age at treatment initiation + Δ disease duration before treatment initiation). For each risdiplam patient, exactly one nusinersen-treated patient with the lowest (best) matching score was selected. Each nusinersen patient was used only once. This process resulted in 29 matched pairs (58 pwSMA in total). Four pairs with an age difference over 15 years were excluded. One pair showed extreme outlier values with unusually high baseline HFMSE and RULM scores and was therefore excluded to improve the statistical integrity and interpretability of our findings.

To compare the differences between the nusinersen and risdiplam groups regarding age at treatment initiation, disease duration until treatment initiation and *SMN2* copy number, a Mann–Whitney U test was applied. To compare the differences between the nusinersen and risdiplam groups regarding sex, functional status, SMA type, and the presence of PEG, ventilatory support, and spondylodesis, a Pearson’s chi-square test was applied. To preserve the matched-pair structure of the sample and keep the analysis robust when some group–time combinations had few observations or limited variability, we chose an analysis approach based on pairwise difference scores (Δ-values). These were calculated by subtracting the risdiplam patient's score from the matched nusinersen patient's score at each time point. This approach allowed for a direct comparison of treatment effects within each matched pair across time. To assess changes over time, a Friedman test was applied to the Δ-values from time points T0 to T4. In accordance with the matched-pair design, pairs were censored pairwise once one patient was no longer available for the respective follow-up. Missing data were mainly due to administrative reasons, treatment discontinuation, switches between therapies, and continuation of care at local centers.

In addition to the paired Δ-value analyses, we performed longitudinal linear mixed-effects models to assess changes in motor scores over time. Patient ID was included as a random effect, while time, treatment, and their interaction were specified as fixed effects. Models were estimated using restricted maximum likelihood (REML) with an autoregressive covariance structure [AR(1)]. Type III tests of fixed effects were applied for statistical inference.

### Results

**Fig. 1:**
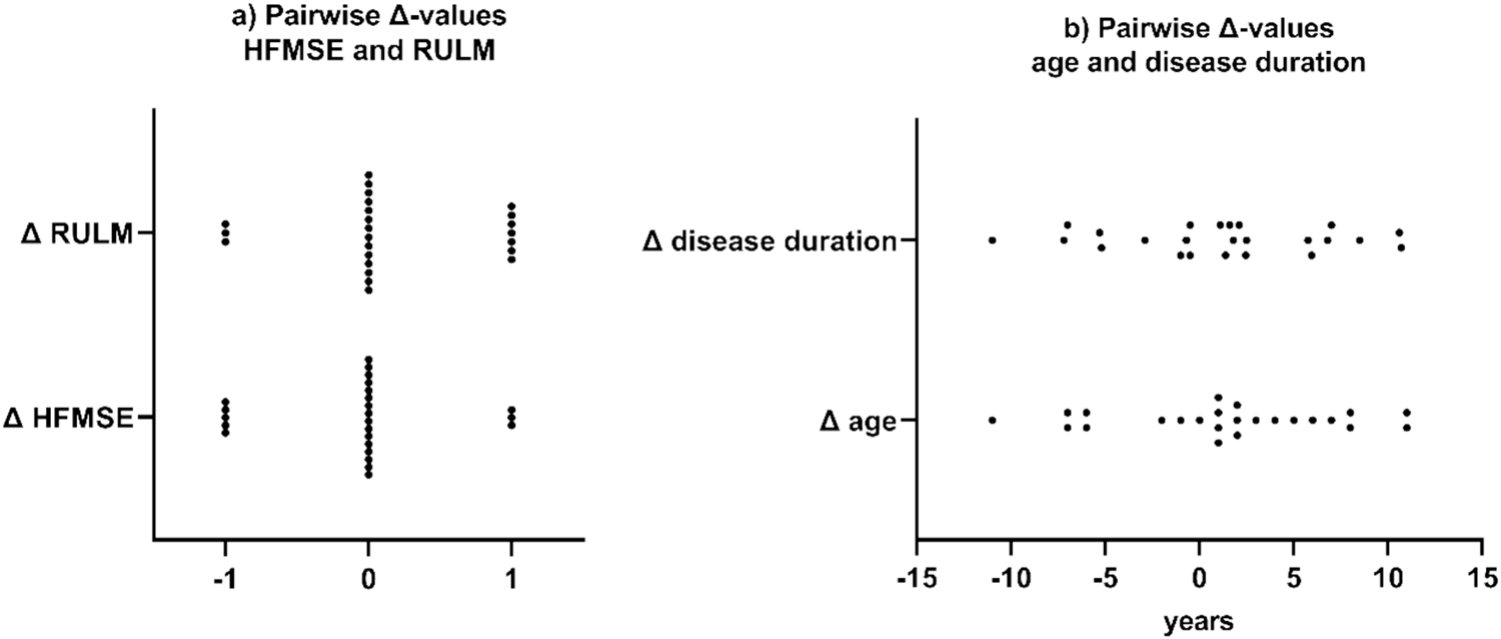
Pairwise Δ-values of **a** HFMSE and RULM and **b** age and disease duration at baseline. Δ-values were calculated by subtracting the score of the risdiplam-treated pwSMA from that of the matched nusinersen-treated pwSMA at the respective time point. Positive Δ-values indicate that the respective nusinersen value was higher than the risdiplam value, whereas negative Δ-values indicate that the risdiplam value was higher than that of the matched nusinersen pwSMA. HFMSE and RULM Δ-values are mostly 0; for RULM, 3 are − 1 and 7 are + 1, and for HFMSE, 5 are − 1 and 3 are + 1. Age and disease duration Δ-values are mostly between − 5 and + 5, with extremes of − 11 and + 11

**Fig. 2:**
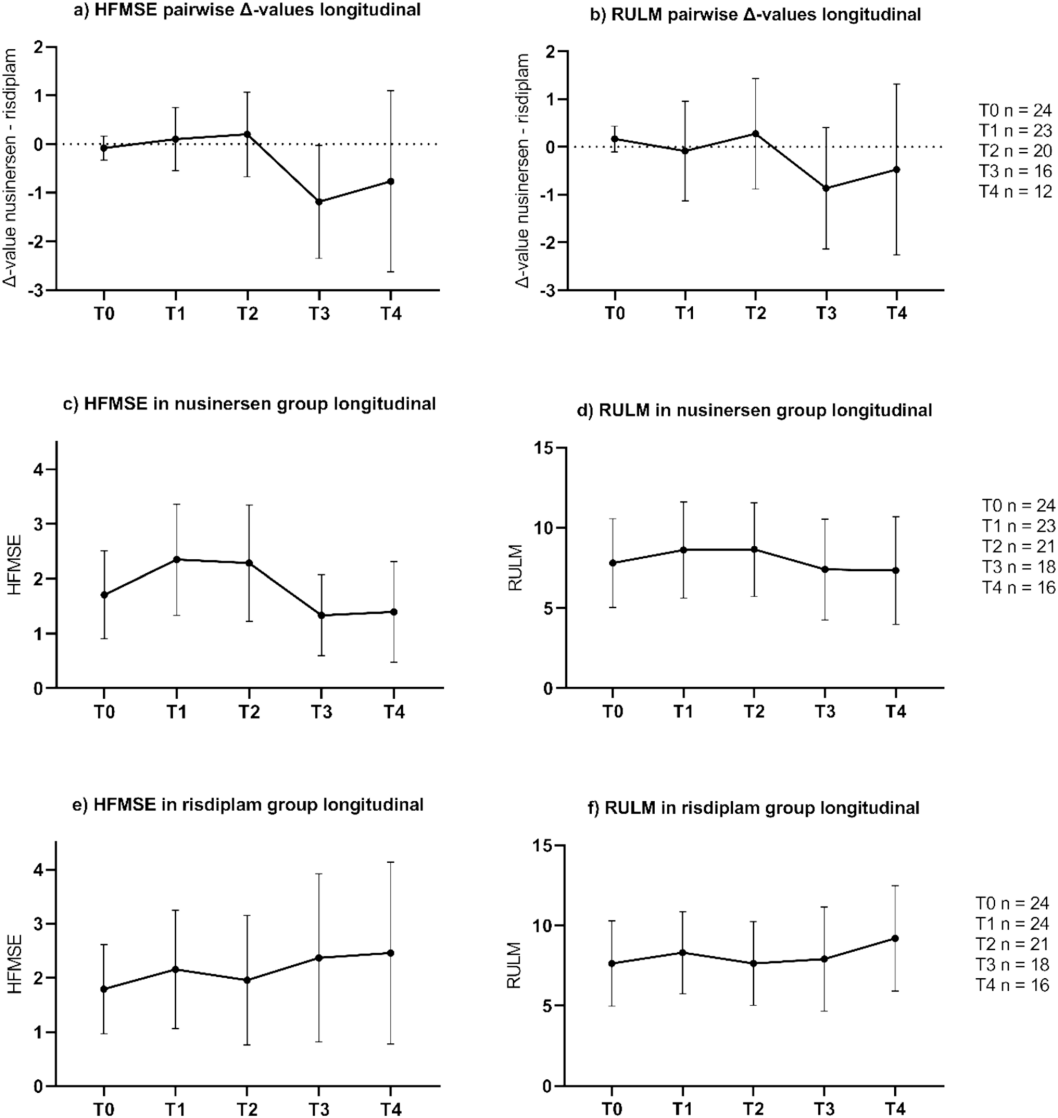
Pairwise Δ-values of **a** HFMSE and **b** RULM scores across time points: baseline (T0), 4–8 months (T1), 10–14 months (T2), 22–26 months (T3), and 32–40 months (T4) after treatment initiation. Δ-values were calculated by subtracting the score of the risdiplam-treated pwSMA from that of the matched nusinersen-treated pwSMA at the respective time point. Positive Δ-values indicate that the respective nusinersen value was higher than the risdiplam value, whereas negative Δ-values indicate that the risdiplam value was higher than that of the matched nusinersen pwSMA. The error bars represent the 95% CI. **c** to **f** show the longitudinal courses of HFMSE and RULM scores in the nusinersen and risdiplam group. The sample size numbers shown on the right refer to each line and represent the number of complete matched pairs available for the paired analyses in (**a**) and (**b**) and the number of individual patients available for analysis in (**c**) to (**f**). Pairwise Δ-values of HFMSE and RULM follow similar trajectories, being close to 0 at T0, T1, and T2 and close to − 1 at T3 and T4

After excluding pwSMA with prior exposure to SMN-targeting therapy and including only those with available HFMSE and RULM scores at baseline and at least at the 4-month follow-up, a cohort of 101 patients was identified: 65 treated with nusinersen and 36 with risdiplam. The demographic and clinical characteristics of this entire, unmatched nusinersen and risdiplam cohort at baseline as well as their group statistics are shown in supplementary Table S1. The final matched cohort, according to the matching process described in the methods, consisted of 24 pairs, corresponding to a total of 48 pwSMA. Within this cohort, age at treatment initiation, disease duration until treatment initiation, sex, SMA type, *SMN2* copy number, functional status, and the presence of spondylodesis, ventilatory support, and PEG did not differ between the nusinersen and risdiplam group. The demographic and clinical characteristics of the matched nusinersen and risdiplam group at baseline are shown in Table 1. The pairwise Δ-values of age at treatment initiation, disease duration until treatment initiation, and HFMSE and RULM baseline scores are shown in Fig. 1. No statistically significant differences in the pairwise Δ-values of HFMSE and RULM were observed across time points T0 to T4. Figure 2 shows the mean pairwise Δ-values of HFMSE and RULM scores across all 24 matched pairs at time points T0 to T4 and the longitudinal course of HFMSE and RULM scores in the nusinersen vs. risdiplam group. Within both treatment groups, there were no significant changes in motor scores from T0 to T4.

**Table 1:** Baseline demographic and clinical characteristics of the matched nusinersen and risdiplam cohorts

	Nusinersen (*n* = 24)	Risdiplam (*n* = 24)
Age, years	34.9 ± 10.4 (21–53)	33.5 ± 12.2 (18–55)
Disease duration, years	33.9 ± 10.1 (20–52)	32.8 ± 12.2 (18–55)
Sex, male	14 (58%)	12 (50%)
SMA type		
Type 1	2 (8%)	1 (4%)
Type 2	13 (54%)	20 (83%)
Type 3	9 (38%)	3 (13%)
SMN2 copy number		
2	2 (8%)	2 (8%)
3	17 (71%)	22 (92%)
≥ 4*	5 (21%)	0 (0%)
Functional status		
Non-Sitter	17 (71%)	18 (75%)
Sitter	7 (29%)	6 (25%)
Spondylodesis	13 (54%)	13 (54%)
Ventilatory support		
Non-invasive nocturnal	13 (54%)	11 (50%)
Non-invasive nocturnal plus daytime use	0 (0%)	1 (4%)
Invasive	1 (4%)	0 (0%)
Percutaneous endoscopic gastrostomy	4 (17%)	1 (4%)
HFMSE score	1.7 ± 1.9 (0–7)	1.8 ± 2.0 (0–7)
RULM score	7.8 ± 6.5 (0–20)	7.6 ± 6.3 (0–20)

Values are presented as mean ± SD (range) or *n* (%)

*Includes 4 patients with 4 copies and 1 with 5 copies

The mixed-effects models confirmed the results of the paired analyses. No significant time × treatment interaction was observed for either HFMSE or RULM, indicating no statistically significant difference in longitudinal trajectories between the nusinersen and risdiplam group within the matched cohort (supplementary Table S2).

In the nusinersen group, missing data were due to incomplete assessments, treatment switches to risdiplam, treatment discontinuation, study participation, and continuation of follow-up at local centers. In the risdiplam group, missing data were due to treatment discontinuation, insufficient follow-up duration at the time of analysis, treatment switches to nusinersen, and continuation of follow-up at local centers. A detailed breakdown of missing data by treatment group and time point is provided in the supplement (supplementary Table S3).

## Discussion

In this comparison of the therapeutic effects on motor function of nusinersen and risdiplam in a matched cohort of adult pwSMA, we found no significant differences in the trajectories of HFMSE and RULM scores over a maximum observation period of almost 3 years. When analyzed separately, neither treatment group showed a significant improvement in motor function as measured by the HFMSE and RULM over the entire observation period. However, there was also no decline in these scores, reflecting the therapeutic effect of both medications against the background of the progressive natural course of the disease [2]. The absence of a difference between the nusinersen and risdiplam group suggests that both medications achieve a similar stabilization of motor function in adults with low baseline function. Our finding is consistent with a study comparing the effects of nusinersen and risdiplam in a cohort of 165 children aged 2–10 years with SMA types 2 and 3, which performed a regression analysis adjusted for baseline motor function as well as other clinical and epidemiological variables, and found no differences in outcomes after 3 and 6 months of treatment [16]. A real-world study examining the switch from nusinersen to risdiplam in 17 patients with SMA aged 3–44 years reported maintained clinical stability and treatment effectiveness after the transition, indicating that risdiplam was not inferior to continued nusinersen therapy [21].

Other real-world studies have shown stabilization or even improvement of motor function in pwSMA treated with nusinersen [11, 22] and risdiplam [13, 14, 23], with, however, only about 10–25% of adult patients achieving a clinically meaningful improvement, defined as a gain of ≥ 3 points in the HFMSE or ≥ 2 points in the RULM. The lack of significant motor improvement in our sample can be explained by the sample characteristics: as noted in the introduction, baseline motor function is on average substantially lower in risdiplam than in nusinersen cohorts [10–14]. This group difference was also evident in our analysis of baseline characteristics of the entire, unmatched cohort, as presented in the Supplement. The matching procedure adjusted the nusinersen group—and thus the entire matched cohort—to this lower baseline, with mean scores of approximately two on the HFMSE and eight on the RULM. It has been shown that patients with lower baseline motor function generally achieve smaller absolute gains under these therapies, whereas groups with higher baseline motor levels have demonstrated sustained long-term motor improvement [10, 24]. Given the low baseline motor function in our sample, only limited change can be expected.

A review on prognostic factors and treatment-effect modifiers in SMA identified disease severity, age at treatment initiation, and disease duration as the most important factors for SMA types 2 and 3 [18]. To achieve the best possible matching of nusinersen/risdiplam pairs, we therefore considered not only baseline motor scores (HFMSE and RULM) but also age at treatment initiation and disease duration until treatment initiation in the matching process. A difference of up to one point in baseline HFMSE or RULM within a pair was permitted to avoid unnecessary reduction of the sample size through overly strict matching as such a small deviation lies within the test–retest variability of the scores [25]. The differences were balanced between treatment groups. Differences in age and disease duration were kept small, with a maximum of 11 years (in three pairs) and were also balanced between treatment groups. As all patients were aged over 18 years with disease durations of 18 years and higher, small age differences are unlikely to have had a relevant prognostic impact.

The matched comparison provides methodologically clear and transparent results by ensuring equivalent baseline levels in key prognostic parameters, thereby supporting causal inference and improving efficiency. Conducting the study at a single center further strengthened the design, as assessments such as motor score evaluations were performed consistently, and therapeutic management beyond drug administration—including recommendations and prescriptions for physiotherapy—was uniform across all pwSMA.

A limitation of the matching approach is that the sample size was restricted by the number of available matched pairs. In addition, the nusinersen group may not fully reflect a typical nusinersen cohort, as it was adjusted to the lower baseline level of the risdiplam group. This restricts the interpretability of the findings, particularly regarding the real-world treatment effect of nusinersen. Furthermore, HFMSE and RULM are often insufficiently sensitive to capture minimal improvements in pwSMA, especially at the lower end of the functional spectrum. A genuine biological effect may therefore have remained below the detection threshold of the scores.

For further comparisons of the effectiveness of nusinersen versus risdiplam, larger sample sizes and longer observation periods would be desirable. With a sufficiently large cohort, it may also become feasible to apply statistical models that account for relevant prognostic factors while still including representative patient samples. This could potentially allow for the identification of specific subgroups that may benefit more from one treatment than from the other.

## Conclusions

In this first matched comparison of nusinersen and risdiplam in adults with SMA, both treatments demonstrated a similar stabilization of motor function over almost 3 years, without significant differences between groups. Given the low baseline motor function of the cohort, substantial improvements were not expected, yet both therapies appeared effective in counteracting the progressive natural course of the disease. Larger and longer-term studies are warranted to confirm these findings and to explore potential subgroup-specific treatment effects.

## Supplementary Information

Below is the link to the electronic supplementary material.

Supplementary file1 (DOCX 20 KB)

## Data Availability

The datasets generated and/or analyzed during the current study are not publicly available due to privacy concerns but are available from the corresponding author on reasonable request.
