# Correction: Matched-pair analysis of motor outcomes in adults with spinal muscular atrophy on nusinersen vs. risdiplam

**DOI:** 10.1007/s00415-026-13669-5

**Published:** 2026-03-28

**Authors:** Svenja Neuhoff, Benjamin Stolte, Jaqueline Lipka, Melina Schlag, Refik Pul, Linda-Isabell Schmitt, Markus Leo, Jelena Skuljec, Cornelius Deuschl, Michael Forsting, Christoph Kleinschnitz, Tim Hagenacker

**Affiliations:** 1 Department of Neurology and Center for Translational Neuro- and Behavioral Sciences (C-TNBS), University Hospital Essen, Hufelandstr. 55, 45147 Essen, Germany; 2 Institute for Diagnostic and Interventional Radiology and Neuroradiology, University Medicine Essen, University of Duisburg-Essen, Essen, Germany

**Correction: Journal of Neurology (2025) 273:55** 10.1007/s00415-025-13589-w

In the original version of this article, the given and family names of all authors were incorrectly structured. The name was displayed correctly in all versions at the time of publication.

The original article has been corrected.

