# Supplementary material for: Matched-pair analysis of motor outcomes in adults with spinal muscular atrophy on nusinersen vs. risdiplam
Source: J Neurol. 2026 Jan 3;273(1):55. doi: 10.1007/s00415-025-13589-w (PMC12764637; doi:10.1007/s00415-025-13589-w)
Supplement: Supplementary file 1 — Supplementary file1 (DOCX 20 KB) [file 415_2025_13589_MOESM1_ESM.docx]

**Supplementary Table S1.** Baseline demographic and clinical characteristics with group statistics of the entire cohort before matching. Values are presented as mean ± SD (range) or n (%).

|  | **Nusinersen (n=65)** | **Risdiplam (n=36)** | **Statistics**^1^ |
| --- | --- | --- | --- |
| **Age, years** | 35.5 ± 12.3 (18–71) | 33.5 ± 11.4 (18–55) | n.s. |
| **Disease duration, years** | 30.0 ± 13.1 (7–67) | 32.3 ± 11.0 (18–55) | n.s. |
| **Sex, male** | 40 (62%) | 16 (44%) | n.s. |
| **SMA type** | | | |
| **Type 1** | 3 (5%) | 1 (3%) | χ²(2) = 33.9,  p < 0.001 |
| **Type 2** | 17 (26%) | 31 (86%) |  |
| **Type 3** | 45 (69%) | 4 (11%) |  |
| **SMN2 Copy Number** | | | |
| **2** | 3 (5%) | 3 (8%) | U = 677.5,  z = −4.13,  p < 0.001 |
| **3** | 33 (51%) | 32 (89%) |  |
| **≥4**^2^ | 29 (44%) | 1 (3%) |  |
| **Functional Status** | | | |
| **Non-Sitter** | 22 (34%) | 24 (67%) | χ²(2) = 16.4,  p < 0.001 |
| **Sitter** | 19 (29%) | 11 (30%) |  |
| **Walker** | 24 (37%) | 1 (3%) |  |
| **Spondylodesis** | 20 (31%) | 20 (56%) | χ²(1) = 5.9,  p = 0.015 |
| **Ventilatory support** | | | |
| **Non-invasive nocturnal** | 17 (26%) | 13 (36%) | n.s. |
| **Non-invasive nocturnal plus daytime use** | 1 (2%) | 2 (6%) | n.s. |
| **Invasive** | 2 (3%) | 0 (0%) | n.s. |
| **Percutaneous Endoscopic Gastrostomy** | 4 (6%) | 1 (3%) | n.s. |
| **HFMSE Score** | 20.0 ± 21.2 (0–62) | 3.5 ± 7.1 (0–42) | U = 662.5,  z = −3.63,  p < 0.001 |
| **RULM Score** | 20.3 ± 13.9 (0–37) | 9.9 ± 7.3 (0–36) | U = 690.0,  z = −3.42,  p < 0.001 |

^1^ Between-group comparisons were performed using χ² and Mann–Whitney U tests, as appropriate. n.s. indicates non-significant results.

^2^Includes 26 patients with 4 copies, 2 with 5 and 1 with 6 copies.

**Supplementary Table S2.** Detailed results of longitudinal linear mixed-effects models assessing changes in motor scores over time.

| **Outcome** | **Effect** | **Numerator df** | **Denominator df** | **F** | **p-value** |
| --- | --- | --- | --- | --- | --- |
| **HFMSE**^1^ | Intercept | 1 | 45.6 | 37.5 | < 0.001 |
|  | Time | 4 | 99.4 | 2.3 | 0.068 |
|  | Treatment | 1 | 45.6 | 0.1 | 0.713 |
|  | Time x Treatment | 4 | 99.5 | 2.3 | 0.062 |
| **RULM**^1^ | Intercept | 1 | 45.9 | 73.3 | < 0.001 |
|  | Time | 4 | 103.2 | 2.1 | 0.089 |
|  | Treatment | 1 | 45.9 | 0.0 | 0.970 |
|  | Time x Treatment | 4 | 103.2 | 0,8 | 0.531 |

^1^Type III tests of fixed effects from linear mixed-effects models for HFMSE and RULM. Numerator and denominator degrees of freedom were estimated using the Satterthwaite approximation. Patient ID was included as a random effect, and time, treatment, and their interaction as fixed effects.

**Supplementary Table S3.** Reasons for missing data at each follow-up time point in the nusinersen and risdiplam group.

| **Time point** | **Nusinersen group**^1^ | **Risdiplam group**^1^ |
| --- | --- | --- |
| **T1** | Incomplete assessment (1) | - |
| **T2** | Switch to Risdiplam (2); Treatment discontinuation (1) | Treatment discontinuation (2); Insufficient follow-up duration (1) |
| **T3** | Switch to risdiplam (3); Treatment discontinuation (1); Continuation of treatment at local centers (1); Participation in a clinical study (1) | Treatment discontinuation (2); Incomplete assessment at T3 (1); Insufficient follow-up duration (2); Continuation of treatment at local centers (1) |
| **T4** | Switch to risdiplam (3); Treatment discontinuation (1); Continuation of treatment at local centers (3); Participation in a clinical study (1) | Treatment discontinuation (1); Insufficient follow-up duration (4); Continuation of treatment at local centers (2); Switch to nusinersen (1) |

^1^Cumulative numbers of missing observations from the respective time point onward are shown in parentheses.
